# *Popillia japonica* – Italian outbreak management

**DOI:** 10.3389/finsc.2023.1175138

**Published:** 2023-05-18

**Authors:** Paola Gotta, Mariangela Ciampitti, Beniamino Cavagna, Giovanni Bosio, Gianni Gilioli, Alberto Alma, Andrea Battisti, Nicola Mori, Giuseppe Mazza, Giulia Torrini, Francesco Paoli, Giacomo Santoiemma, Anna Simonetto, Federico Lessio, Giorgio Sperandio, Emanuela Giacometto, Alessandro Bianchi, Pio Federico Roversi, Leonardo Marianelli

**Affiliations:** ^1^ Settore Fitosanitario e servizi tecnico-scientifici – Piedmont Region, Turin, Italy; ^2^ Directorate General (DG) Agricoltura Servizio Fitosanitario Regionale, Lombardy Region, Milan, Italy; ^3^ Dipartimento di Ingegneria Civile Ambiente Territorio Architettura e Matematica (DICATAM), University of Brescia, Brescia, Italy; ^4^ Dipartimento di Scienze Agrarie, Forestali e Alimentari (DISAFA), University of Turin, Turin, Italy; ^5^ Department of Agronomy, Food, Natural Resources, Animals and the Environment (DAFNAE), University of Padua, Padua, Italy; ^6^ Department of Biotechnology, University of Verona, Verona, Italy; ^7^ Council for Agricultural Research and Economics (CREA) - Research Centre for Plant Protection and Certification, Florence, Italy

**Keywords:** biological control agents, biological invasion, chemical control, EU priority pest, Japanese beetle, pest management, spatial analysis

## Abstract

*Popillia japonica*, a priority pest for the EU, was first detected in Northern Italy in 2014. Since its discovery, the outbreak extended over an area of more than 16,000 square kilometers in Northern Italy and Southern Switzerland. In this review, we summarize the state-of-the-art of research conducted in Italy on both the spreading capacity and control measures of *P. japonica.* Chemical, physical, and biological control measures deployed since its detection are presented, by highlighting their strengths and weaknesses. An in-depth study of the ecosystems invaded by *P. japonica* disclosed the presence and pathogenicity of natural strains of entomopathogenic fungi and nematodes, some of which have shown to be particularly aggressive towards the larvae of this pest under laboratory conditions. The Plant Health authorities of the Lombardy and Piedmont regions, with the support of several research institutions, played a crucial role in the initial eradication attempt and subsequently in containing the spread of *P. japonica*. Control measures were performed in the infested area to suppress adult populations of *P. japonica* by installing several traps (e.g., for mass trapping, for auto-dissemination of the fungus *Metarhizium anisopliae*, and “attract & kill”). For larval control, the infested fields were treated with commercial strains of the entomopathogenic fungus *M. anisopliae* and nematode *Heterorhabditis bacteriophora*. Future studies will aim at integrating phenological and spread models developed with the most effective control measures, within an ecologically sustainable approach.

## 1 Introduction

The Japanese beetle, *Popillia japonica* Newman, 1841 (Coleoptera: Scarabaeidae) is a scarab beetle native to Japan and known to be a pest of agricultural crops, turfs, ornamental and forest plants in the introduction areas. The beetle has more than 300 host plants and is a strong flier, in addition, it has a great ecological plasticity that allows it to invade large areas in a short time (1–4).

**Figure 1:**
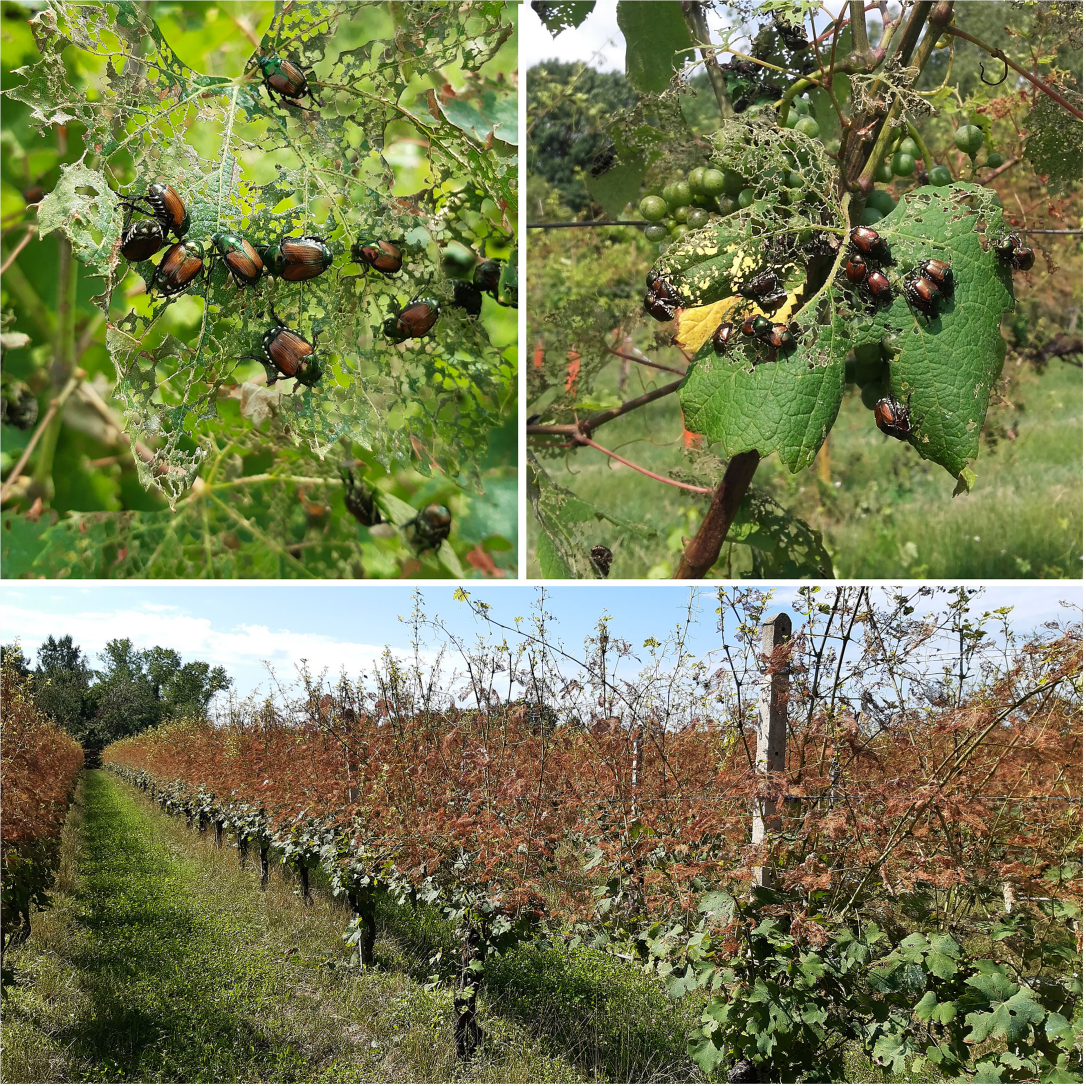
Damage caused by adults of *Popillia japonica* on vineyards.

*Popillia japonica* was introduced in 1916 in the US (1) and, within 100 years, it colonized most of the eastern and central US territories along with a part of eastern and western Canada (5). In Europe, *P. japonica* was detected for the first time in the early 1970s on Terceira Island (Azores archipelago). Since its first discovery, phytosanitary measures have been applied to manage *P. japonica*, such as chemicals, mass trapping, and biological control agents (6–10). Nevertheless, over the past 50 years, the infestation has reached other Azores islands: Faial, Flores, Pico, São Jorge, São Miguel, and Graciosa (11, 12). Due to its spread capacity and the potential impact on crops, *P. japonica* has been included in the European Plant Protection Organization (EPPO) list A2 as a quarantine pest recommended for regulation (https://www.eppo.int/ACTIVITIES/plant_quarantine/A2_list). In 2019, *P. japonica* was listed as a priority pest (13) and classified as the second most crucial potential priority pest in Europe (14, 15). In July 2014, a wildlife photographer posted a photo of *P. japonica* on the naturalist forum “Natura Mediterraneo” (https://www.naturamediterraneo.com/forum/). This was the first report of this pest in north-western Italy and in mainland Europe (16). The Ticino River valley, a Natural Park between the Lombardy and Piedmont regions, located in Northern Italy, was soon identified as the outbreak area as Plant Health authorities of the Lombardy and Piedmont regions (parts of the National Plant Protection Organization) detected a high-density of larvae, which locally exceeded 300 individuals per square meter. This high level of infestation attracted many vertebrate larval predators such as birds, moles, and wild boars, which resulted in further damage to the turf. However, the major impacts on crops, such as vineyards (**Figure 1**), soybean, and corn, were caused by adults feeding on leaves and fruits. Furthermore, the damage was also observed in private orchards of peach, plum, apple, persimmon, and other types of fruit plants present in the infested areas. Defoliation also involved vegetables and ornamental plants (17). The Plant Health authorities of the Lombardy and Piedmont regions reacted promptly, and several phytosanitary measures were adopted to monitor the area and to contain the *P. japonica* populations. Visual inspections and control measures were focused on all high-risk sites, e.g., plant nurseries and sites suitable for passive dispersal, such as industrial areas, trucking companies, railway stations, sports fields, playgrounds, boulevards, waste collection areas, petrol stations, car and truck parking areas, swimming pools, and shopping centers, considering the hitchhiking behavior of *P. japonica* adults (5, 18). Control measures were performed in the infested area to suppress adult populations of *P. japonica* by installing several double-baited traps that attract both sexes using a floral attractant and a synthetic pheromone: i) funnel traps for mass trapping, ii) traps for auto-dissemination of the fungus *Metarhizium anisopliae* (Metch.) Sorok, 1883, and iii) “attract & kill” devices containing alpha-cypermethrin or deltamethrin (19, 20). For larval control, 2,200 ha were treated with biological control agents such as the entomopathogenic nematode *Heterorhabditis bacteriophora* (Poinar, 1975) (Nematoda: Rhabditidae) and the entomopathogenic fungus *M. anisopliae* (21). Specific risk-based plans and peculiar prescriptions were provided in the airport and cargo areas of Malpensa and Cameri (respectively in the Lombardy and Piedmont regions), located within the infested area, to avoid the spread of *P. japonica* adults from an infested to pest-free area.

**Figure 2:**
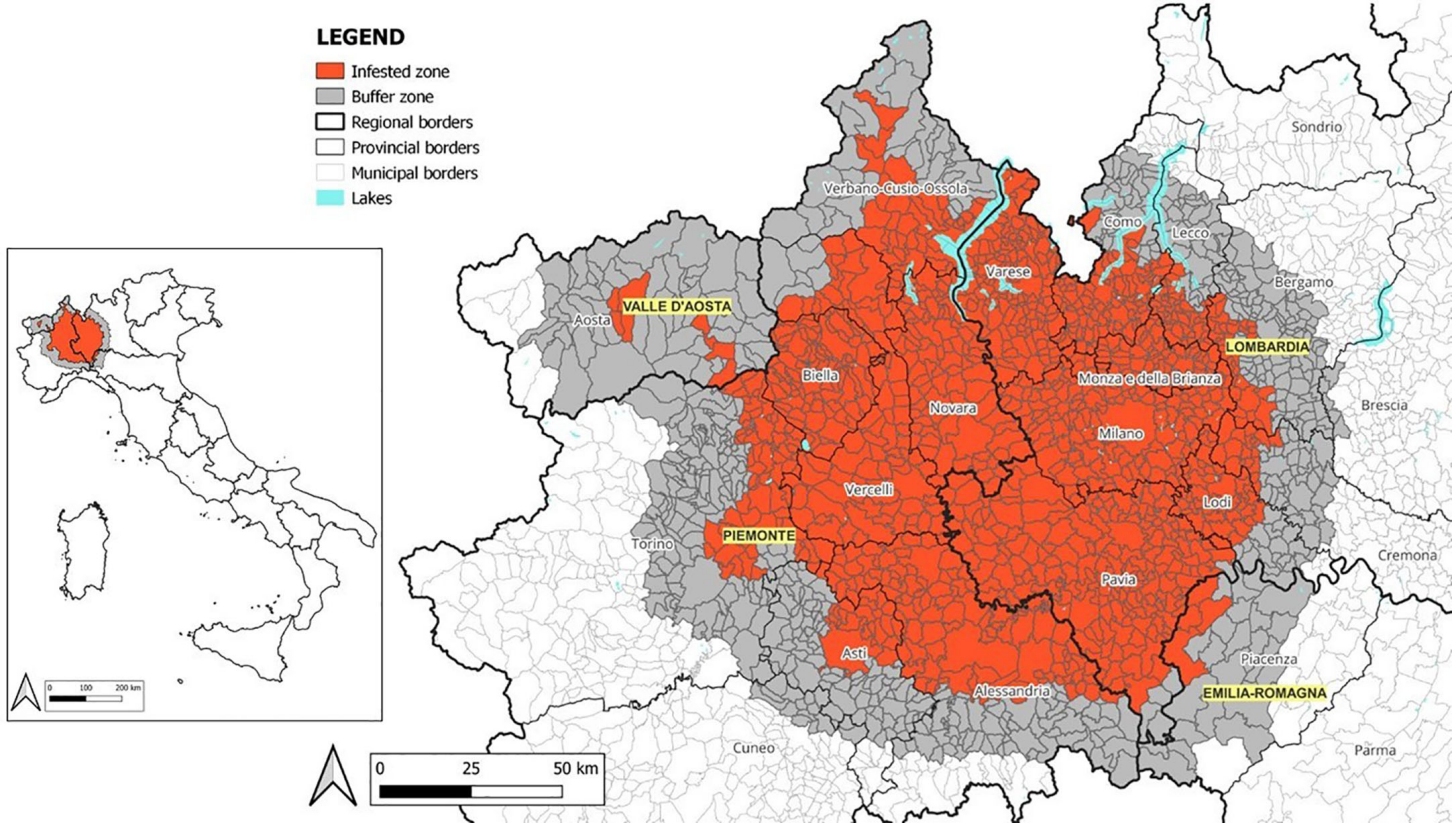
Italian demarcated area of *Popillia japonica*.

The huge efforts made to cope with *P. japonica* limited the spread of this pest in Italy, which reached 16,232 km² (Europhyt Outbreak No. 574, Update 07/2022-12-05) (**Figure 2**). Nevertheless, the pest was established also in the contiguous area of Ticino in Southern Switzerland (22), while interceptions outside the current area of infestation were reported in the Netherlands (23), Germany (24), and Italy (25, 26).

Since the first year of discovery, several research activities were conducted by the Plant Health authorities of the Lombardy and Piedmont regions and national research institutions (University of Brescia, Turin, Padua and Verona, and CREA-Research Centre for Plant Protection and Certification of Florence), to understand the ecology of the pest in the new introduction range and to contain larval and adult populations.

Here, we reviewed and summarized the knowledge on the processes determining the potential spread of *P. japonica* in recently invaded areas in Italy. In addition, we reviewed the chemical, physical, and biological control methods adopted against this pest, by considering their pros and cons. Finally, we pinpoint management techniques to be considered for the development of integrated control strategies.

## 2 Potential spread of *Popillia japonica* on the Italian territory

### 2.1 Prevention of the spread of *Popillia japonica* in Italy

Nurseries are one of the highest risk sites and therefore deserve a system of prescription and prevention measures to exclude the presence of *P. japonica*. Notwithstanding the movement of plants with soil, from infested to pest-free areas is banned, the Plant Health authorities of the Lombardy and Piedmont regions may authorize the movement if the plants have been grown in a pest-free production site of a registered operator, as defined by the Regulation (EU) 2016/2031 (art. 65) (27), subjected to official inspections in compliance with the Commission Implementing Regulation (EU) 2019/66 (28) and the Commission Implementing Regulation (EU) 2021/2285 (29) for the detection of *P. japonica*. However, one of the following requirements must be met to avoid the passive transport of *P. japonica*: i) the plants must be grown in sites with complete physical protection; ii) any soil residuals that could harbor juvenile stages have to be removed; iii) only commercial potting soil subjected to an insecticide soil treatment is used. The reuse of potting soil is possible only after a heat treatment at 49°C for at least 15 minutes. Concerning the field-grown plants, some precautions should be applied such as: i) milling the soil at least four times, at a depth of 15 cm, ii) treating the soil with insecticides, and iii) removing, for large plants, the first 20 cm of soil.

### 2.2 Spread of *Popillia japonica*

The study of the invasion process is essential to ensure the implementation of proper management plans for both the eradication and the containment of invasive species. The development and use of different spatial approaches to study the spread and support the management of invasive species have been well covered over the past 15 years (30–32). Surprisingly, the spatial dynamics of *P. japonica* have received little attention. Some authors have focused on investigating the local patchy distribution of both larvae and adults (33–36) or the flight activity of the species (4, 37). However, the assessment of the population spread of *P. japonica* at the landscape scale is less represented (38, 39).

This chapter explores and summarizes the available knowledge on the processes determining the potential spread of *P. japonica* in newly invaded areas, particularly the results of the data analysis and modeling tools proposed for describing pest spread in Northern Italy. We considered three fundamental steps in the spatial spread of *P. japonica*: i) the dispersal process, ii) the pattern of population growth, and iii) the rate of population spread.

#### 2.2.1 Dispersal

The pattern of dispersal of *P. japonica* can be investigated by considering two main components: i) individual short-distance dispersal based on random movement or some sort of guided flight based on cues related both to food resources (for both males and females) and to mate finding (only for males), and ii) the occurrence of a discrete event of long-distance dispersal based on natural means or due to human-assisted transportation.

The individual dispersal capacity of *P. japonica* is highly influenced by several parameters. Cloud cover, strong winds (above 20 km/h), or suboptimal temperatures (above or below 25.5°C) (37), in addition to land management practices such as intercropping systems (40, 41) or the application of pesticide treatments (42), significantly reduce the flight activity. In Italy, the main flight activity of *P. japonica* is known to occur between 12:00 pm and 3:00 pm in summer with low levels of relative humidity (43). This pest can fly an average distance of about 2.3 km in 24 hours, with up to 12 km in some cases, as demonstrated by means of a mark-capture technique (4). *Popillia japonica* adults show strong gregarious behavior, which facilitates finding food sources and/or mates. Mated females commonly represent the pioneers that colonize novel areas. Then, both sexual and feeding-induced odor attractants cause aggregation by the joiner individuals, providing further mating opportunities to the females, which have to mate more than once in their lifetime (2, 35).

Long-distance dispersal through the movement of infested soil and/or plants for planting or hitchhiking is also possible for *P. japonica*. The occurrence of long-distance spread events has also been documented outside the global infestation areas (Germany and the Netherlands) (23, 24). Between 2021 and 2022, new incursions of *P. japonica* adults were also reported in other parts of Italy such as Sardinia and Friuli Venezia Giulia regions (25, 26). These events could start new infestation *foci* from which a continuous spread process can originate. The consequence of stratified dispersal (44), summed to the continuous spread with discrete and long jumps events is poorly investigated for *P. japonica* but, as for other species, we expect it will be responsible for the establishment of an exponential pattern of growth in the infested area (45). To manage the risk associated with long-distance dispersal, various measures (including monitoring, removal of host plants, and treatments) are applied in high-risk areas such as big parking areas, loading and unloading docks, refueling stations, ports, and airports (46).

#### 2.2.2 Population growth

**Figure 3:**
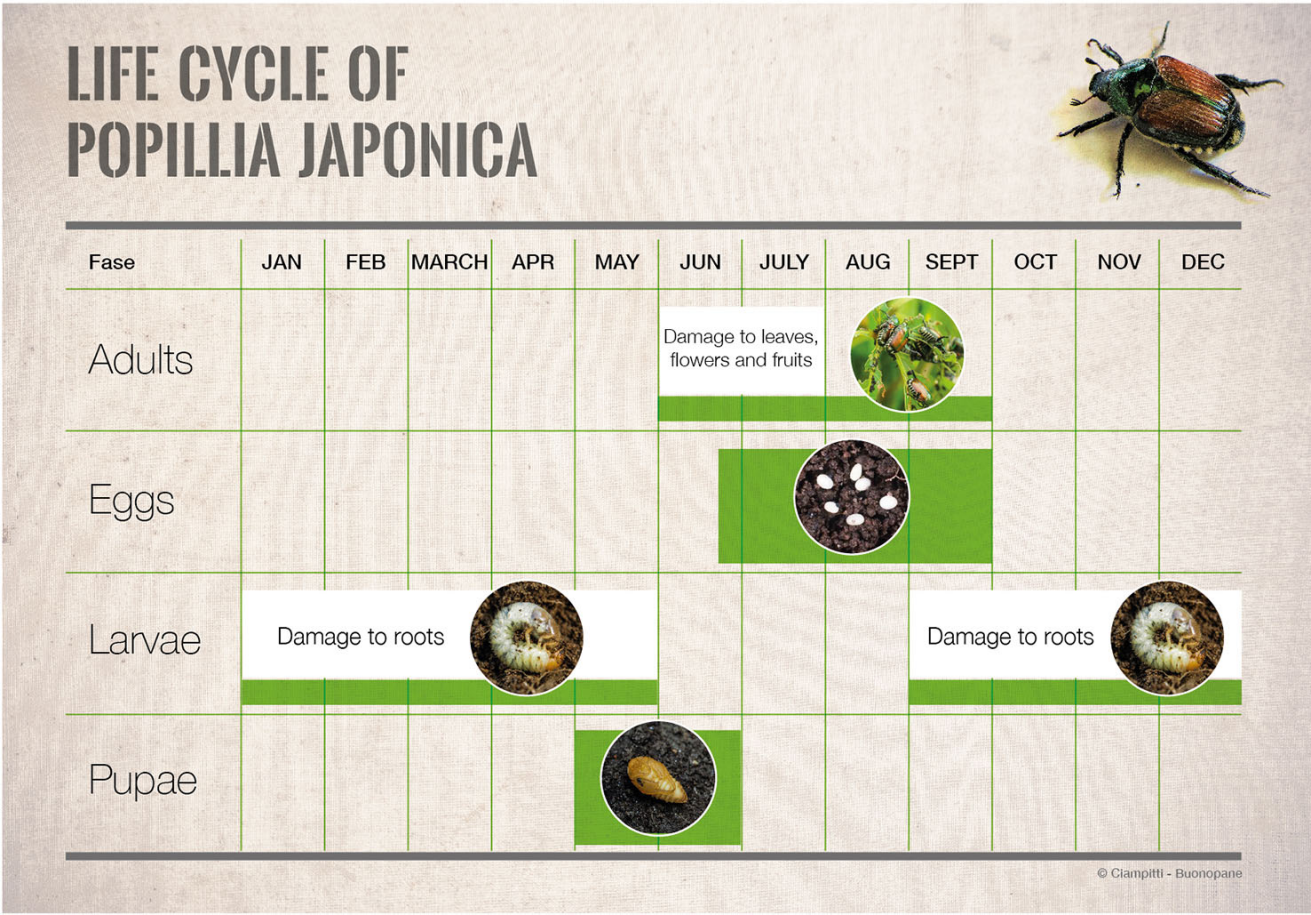
Life cycle of *Popillia japonica* in the Italian outbreak.

The continuous spread of a pest can be considered the result of both individual dispersal and population growth (44). Therefore, knowledge about the population growth of *P. japonica* is fundamental for better understanding the invasion process occurring in Northern Italy and for planning and implementing appropriate control measures (47, 48). The population growth pattern of *P. japonica* was investigated during the ongoing invasion process occurring in Northern Italy. Monitoring data on larvae (through soil cores) and adults (using Trécé^™^ traps baited with dual semiochemical lure that attracts both sexes) collected since 2015 in the Lombardy region were analyzed to predict both the phenological patterns of the species in the infested area (49) (**Figure 3**) and its population growth pattern over the years of infestation. The latter was estimated using a time-discrete logistic model (the Beverton-Holt model, see 50) on the available adult population abundance data collected through Trécé^™^ traps. The results showed that, during the first years of infestation in a novel area, *P. japonica* population abundance is rather low, making the early detection of the pest relatively difficult. After this initial phase, in case the area is suitable for the presence of *P. japonica*, population build-up is rather prominent following a logistic-type (i.e., sigmoid) pattern that leads to high adult population density (on average, the daily adult population abundance reaches 200-300 individuals per trap per day after 4-5 years since the first infestation). Model predictions show that *P. japonica* reaches the maximum population abundance after 7-8 years since the first outbreak.

#### 2.2.3 Population spread

Most of the available knowledge on *P. japonica* spread comes from studies conducted in the US. These studies report varying results, depending on the time elapsed since the first infestation and the overall suitability of the investigated area. In Smith and Hadley (51), a spread rate ranging from 16 to 24 km/year was reported a decade after the first infestation of *P. japonica*. Fox (52) later reported a spread rate ranging from 3 to 24 km/year. Allsopp (38) reported that the spread rate of *P. japonica* was increasing over time, being 7.7 km/year between 1927 and 1938, and 11.9 km/year between 1939 to 1951.

Different approaches and modeling tools were applied to estimate and predict the spread rate of *P. japonica* populations, and to explain the role of relevant variables influencing the spreading process. Mondino et al. (39) proposed an iterative spatially-based model to interpret and forecast the spreading dynamics of *P. japonica*. Five years of trapping data were used for model parametrization (2015-2016) and validation (2017–2019). Variograms from both trapping data and model simulations suggested a range of spatial autocorrelation ranging from 7.5 to 15 km (with a determination coefficient (R^2^) ranging between 0.39 and 0.87). The model proposed by Mondino et al. (39) includes a source dilution factor describing the rate of *P. japonica* moving away from a given position, based upon variograms, a parameter quantifying the increase in *P. japonica* population at the same location over years, and a mortality factor estimated based on literature data. Mondino et al. (39) also considered the role of land cover in determining the probability of colonization of new areas. Prediction maps indicate a potential spread of *P. japonica* mainly southwards and southeastwards, matching the distribution of suitable land covers (e.g., meadows, croplands, woods).

With the comprehensive monitoring dataset (from 2015 to 2021) on the time-series adult catches of the infested areas in the Lombardy region, the spatial and temporal dynamics of *P. japonica* population abundance were investigated using a discrete-time continuous-space reaction-diffusion model (50, 53). This analysis allowed the confirmation that the speed of invasion varies over different spatial directions starting from the area of the establishment. To better understand the spreading process and the factors affecting the speed of invasion, 14 different spreading trajectories were analyzed in that area. The estimated speed of the traveling fronts showed a fair degree of variability (coefficient of variation = 34%) with a maximum speed higher than 13 km/year and a minimum speed lower than 5.5 km/year, 2.5 times less than the maximum. The analysis showed that the suitability of the habitats plays an important role in determining the speed of the traveling fronts. In the fastest-moving directions, more than 80% of the habitats are suitable for the presence of pest (e.g., arable land, perennial meadows, broadleaf forests, urban green areas, rice fields, or agricultural woodlands). In the slower speed trajectories, over 40% of the habitats are not suitable for the pest (e.g., coniferous or mixed forests, or non-vegetated urban areas). From preliminary estimates, it appears that the traveling front speed increases by about 1.5 km/year for every 10% increase in areas suitable for *P. japonica*. The data analysis on larval abundance from soil samples (from 2015 to 2021 of the infested areas in the Lombardy region) showed that *P. japonica* prefers soils that are not loamy sand or acidic. In particular, the relation of pH on species’ suitability shows a non-linear trend and it seems strongly influenced by soil particle size (54–56). Soils with a medium organic matter content are preferred by *P. japonica*, rather than soils with high or low organic matter content (33, 56, 57). Results also showed a non-linear trend between habitat suitability and soil humidity, with extremely dry (58, 59) and relatively wet soils (56) being not suitable for the pest. Similarly, extremely low (below 13°C) or high (above 34°C) temperatures are not suitable for the pest (2, 56, 58, 59). The role of climate and climate change on the potential distribution of *P. japonica* has been studied by Zhu et al. (60) and Kistner-Thomas (59). Changing temperatures are expected to favor the occurrence of the pest in areas above 37° N latitude. Global warming is also expected to increase the area where the species is able to complete a single generation per year, especially in areas above 37° N latitude (59). These effects could lead to an increase in the population abundance of *P. japonica* in the northernmost areas of its range, which could have a positive effect on the population growth and spread rate of the pest (44). In contrast, areas below 8° N latitude are expected to become less suitable for the pest due to an increase in temperature above the optimal range (59).

## 3 *Popillia japonica* outbreak management

### 3.1 Chemical control

In the early stages of the spread of an invasive pest, the use of insecticides is critical to meet immediate pest management needs. The use of broad-spectrum insecticides to control growing *P. japonica* populations was widely used in the early years of the last century in the US due to their effectiveness and relatively low cost (2, 61, 62). Problems caused to non-target insect populations, as well as to human health, and other warm-blooded animals, have curtailed their use and changed spraying methods (5).

Extensive EU legislation regulates the marketing (Regulation (CE) 2009/1107) (63), and use (Directive 2009/128/EC) (64) of plant protection products and their residues in foodstuffs. While this regulation reduces the environmental risks of the pesticide, it negatively affects the availability of active ingredients (AIs) for *P. japonica* containment. The current limitation of products registered against this species required experimental tests to evaluate the effectiveness of the AIs available in European countries.

In 2017-2019, field trials were carried out in vineyards in Novara (North-eastern Piedmont) testing chemical insecticides and organic products with repellent and/or phago-deterrent effects against *P. japonica* adults. The effectiveness of the different substances was evaluated by counting the adults before and after treatments and by estimating the defoliation rates in the different plots. Deltamethrin, etofenprox, lambda-cyhalothrin, acetamiprid, and chlorantraniliprole showed a high-medium efficacy in reducing adult infestations and defoliation rates, while tau-fluvalinate, chlorpyrifos-methyl, and organic pyrethrins had lower activity. Among the repellent/phago-deterrent substances, neem oil and zeolite (chabazite) were quite ineffective, while kaolin clay reduced the number of adults feeding on the vines (65). Additionally, the side effects on phytoseiid mites were evaluated by inspecting leaf samples in the laboratory under a stereomicroscope, but only pyrethroids negatively affected the predatory mite populations (65).

In 2019 and 2020, 20 AIs representative of chemical and organic insecticides registered in Europe for the management of adult beetles were tested. The trials were carried out at five sites located in the infested area (Milano and Varese provinces). The target plant species were three high-value crops (grapevine *Vitis vinifera* L., peach *Prunus persica* (L.) Batsch, and corn *Zea mays* L.) and two landscape plants (goat willow *Salix caprea* L. and Virginia creeper *Parthenocissus quinquefolia* (L.) Planch.). For each site, branches were covered with a protective net (70 × 100 cm, mesh 1 × 1 mm) and 25 adult beetles were introduced. Three experimental conditions were tested for each insecticide: contact, short-term, and long-term (residual) effects. Beetles were introduced before the spraying (contact), right after (short-term), and one week after (long-term) (66). Four replicates in each experimental condition and site were used and insect mortality was assessed until three weeks after the treatment. Acetamiprid, deltamethrin, lambda-cyhalothrin, and phosmet, which are broad-spectrum insecticides, showed to be effective in killing beetles under all experimental conditions. Most of the other AIs were effective only by contact and short-term residual. The organic AIs were not effective under any condition; a mixture of an organic ingredient, paraffinic mineral oil, with cypermethrin showed good efficacy on contact (66, 67).

**Figure 4:**
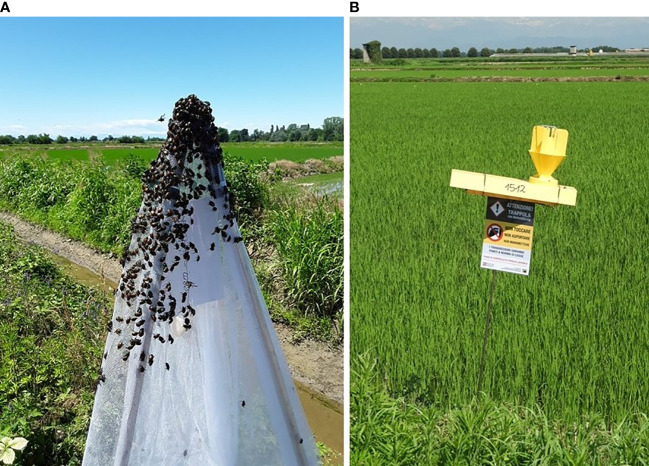
**(A)** Attract & kill device with long-lasting insecticide-treated net. (LLIN) The LLIN is mounted on a tripod frame made of telescopic tubes extendable up to 2 m in height **(B)** Traps (H: 35 cm, L: 60 cm, W: 18 cm) for auto-dissemination of the fungus *Metarhizium anisopliae*.

The lack of long-term efficacy of the insecticides registered in Europe for adult management led to applications repeated many times during *P. japonica* flight period, starting from adult emergence (65). Moreover, the low selectivity of the effective AIs suggests integrating these chemicals into a general management plan that envisages the use of these pesticides only where necessary. To limit the adoption of chemical products and preserve non-target species in the environment, the use of Long-Lasting Insecticide-treated Nets (LLINs) activated with pyrethroids has been tested since 2017 (68). This technology was originally conceived to protect people from vector-borne diseases, such as malaria or yellow fever, and then its use has been extended to agricultural management (69–75). To protect crops and stored products from *P. japonica* attract-and-kill devices (frames containing floral attractant and synthetic pheromone lures), covered with LLINs (usually made of polyester or polyethylene fibers) impregnated with or coated by insecticides such as alfa-cypermethrin or deltamethrin at concentrations ranging from 1 to 4 mg AI/g fiber, are being used (**Figure 4A**). In this way, *P. japonica* adults are attracted by the lure, get in touch with the LLIN and eventually die. To evaluate the effectiveness of LLINs against *P. japonica*, laboratory tests were performed with adult *P. japonica*. The insects (both males and females) were allowed to walk on the LLIN at different exposure times. At the end of the experiment, mortality ranged from 89% to 100% for alfa-cypermethrin, while 100% mortality was observed for deltamethrin irrespective of the exposure time (68). An evaluation of how long alfa-cypermethrin LLIN can be effective during the flight season was also carried out. As a result, the mortality of *P. japonica* decreased by about 30% after one month of field exposure, although still significantly different from the control. After two and three months of field exposure, the mortality was similar to that of the control (43). This is not unexpected because pyrethroids are known to decay under sunlight exposure (76), and so does their killing effectiveness. LLINs were deployed on a large scale by the Plant Health authorities of the Lombardy and Piedmont regions to control this pest in the infested area.

#### 3.1.1 Experimental soil-injection machine

To control *P. japonica* larvae in the soil, an experimental soil-injection machine (“Eco Defender 25”), was conceived and produced in collaboration with the company “MA/AG” and tested in two highly infested perennial meadows in the Lombardy region. The machine injects liquid solutions into the soil with minimum turf damage and soil agronomic characteristics perturbation. At the end of the summer of 2020 and 2021, biological control agents (entomopathogenic nematodes-EPNs, and fungi-EPF) and the insecticide Acelepryn (AI chlorantraniliprole at 20%), authorized for turf application by derogation in accordance with Regulation (CE) 2009/1107 (art. 53) (63), were applied. The effects of the products were evaluated by counting the number of live larvae in the soil after 40 days of the application. There was a general reduction of larval density in the treated plots, with the Acelepryn formulation performing better in the short term (67).

### 3.2 Physical control

#### 3.2.1 Weed mulching products and humidity control on potted plants

**Figure 5:**
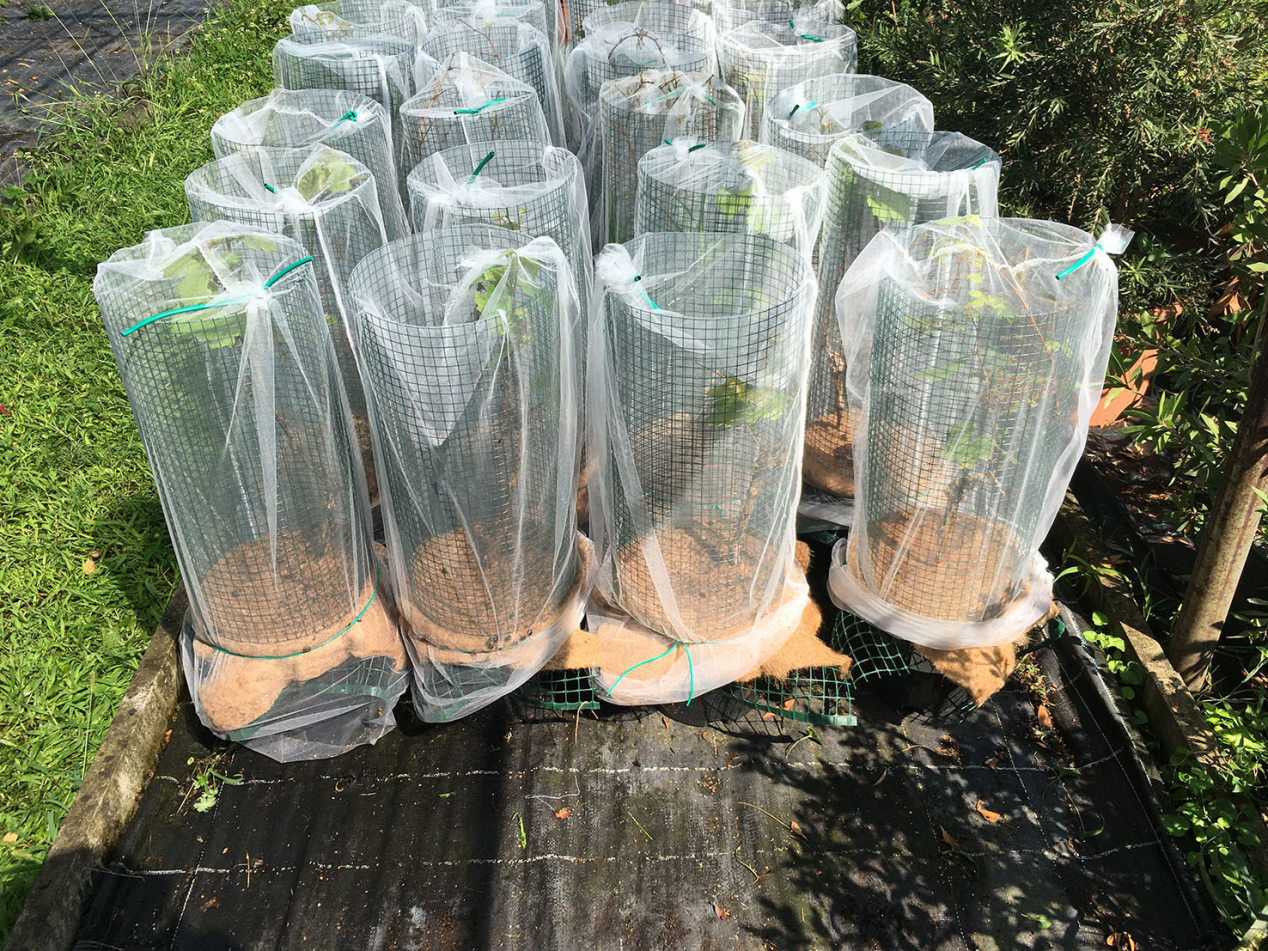
Trial of physical barriers deployed above the soil in containers for ornamental plants in a nursery.

Due to the restrictions on transporting plants/soil from areas infested with *P. japonica*, a new approach was used to prevent beetle oviposition in grapevine potted plants. In 2020, in a nursery located in the infested area of the Lombardy region, three commercial weed mulching products for potted plants were used to assess the oviposition ability of female *P. japonica*. The tested weed mulching products were coconut fiber mulching discs (two densities), jute fabric, and wood chips (**Figure 5**). Results showed that coconut mulching was able to strongly reduce oviposition and further larval development (67, 77).

In 2021, in the same nursery, the survival of *P. japonica* larvae was challenged by applying a mild water stress treatment to potted plants. In the pots subjected to daily irrigation, the average number of larvae found was about five times higher than that in water-stressed pots. At the end of the experiment, both daily irrigated and mildly stressed plants returned to normal conditions (78).

#### 3.2.2 Nets barrier

To protect nursery plants from adult insect damage, in a heavily infested nursery located in the Lombardy region, three net types were tested as physical barriers to protect grapevine potted plants from *P. japonica* adults. The types of nets were anti-hail nets, anti-hail nets treated with permethrin, and insect-proof nets. All tested nets were equally able to protect the plants from adult beetles for the entire duration of the trial compared to those with no protection. The use of an anti-hail net could be the best option to protect nursery plants given the lower costs compared with an insect-proof net (79).

## 4 Biological control

**Figure 6:**
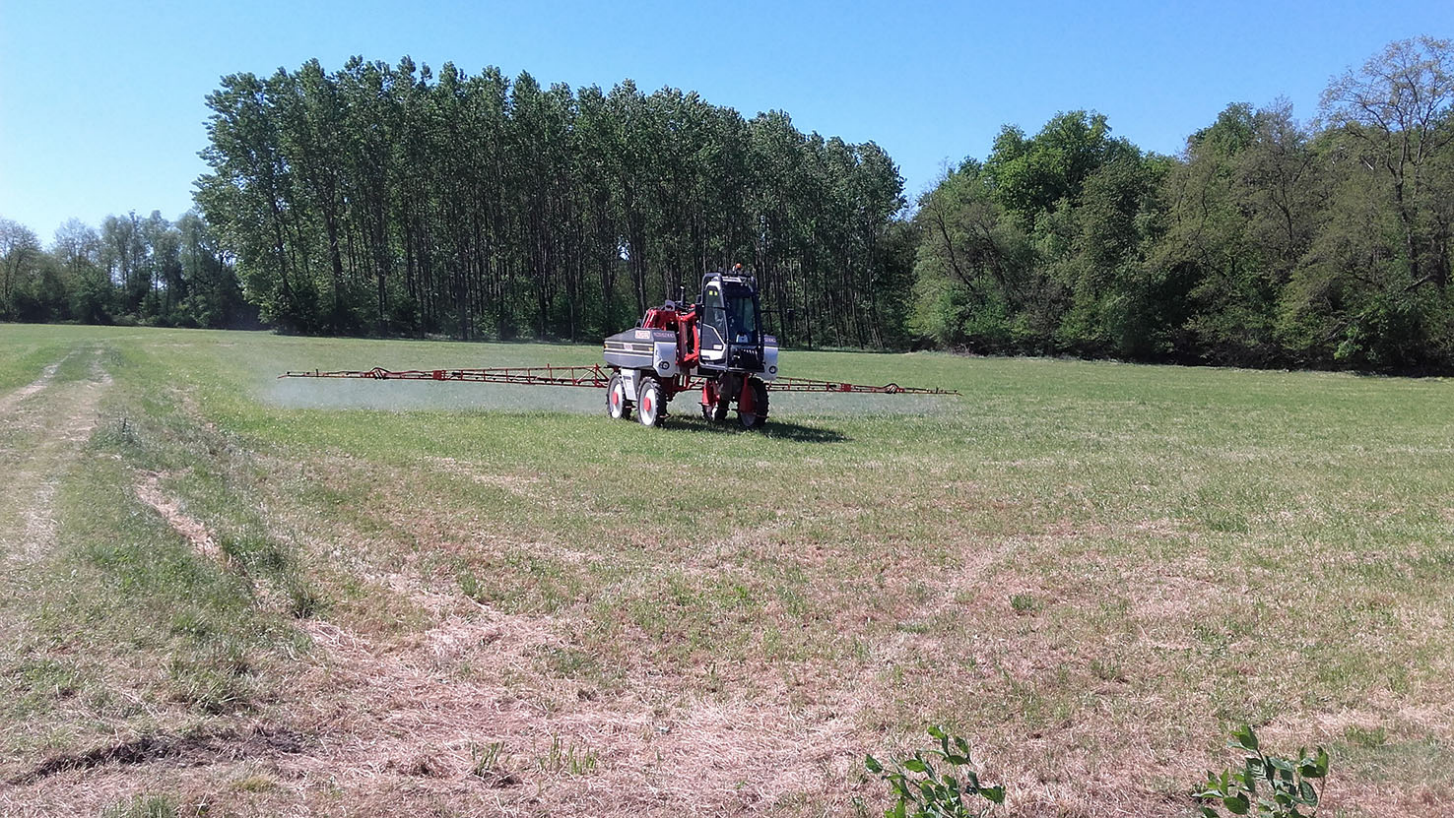
Treatments with entomopathogenic nematodes (EPN) in fields infested with *Popillia japonica* larvae.

**Figure 7:**
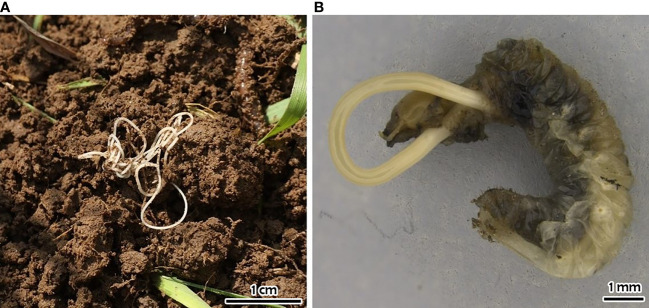
*Hexamermis popilliae*: **(A)** free living specimen *sensu* Mazza et al. (92); **(B)** third-stage postparasitic juvenile emerging from *Popillia japonica* larva.

Several methods have been developed to control *P. japonica* and environmentally friendly strategies, based on the use of natural enemies, have been reported since 1920 (e.g., 80, 81), and tested also in Italy. Many EPF are relatively common worldwide, often trigger epizootics, and can therefore be considered an important factor in controlling insect populations. Several EPF (*Metarhizium* spp. Sorok. and *Beauveria* sp. Vuill.) have also been tested against larvae, pupae, and adults of *P. japonica* in laboratory, semi-field, and field trials worldwide but with contrasting results (2). At the beginning of the Italian invasion, different species of commercial EPF were tested in both controlled and open-field trials. *Beauveria bassiana* (Bals.-Criv.) Vuill. (1912) (commercial strain ATCC 74040) was tested against adults in an ornamental plant nursery in Northern Italy, but the treatment was found to be ineffective (67). The genus *Metarhizium*, on the other hand, has been evaluated several times. Benvenuti et al. (82) tested the commercial *Metarhizium brunneum* Petch, 1939 strain BIPESCO5 against pupae in semi-field applications and found an overall adult mortality of about 40% in 12 days. Benvenuti et al. (83) tested an experimental auto-disseminating device (**Figure 4B**) that attracts, infects, and releases adults into the environment to spread the EPF to healthy populations. The device was activated with the two commercial products available in Italy (GranMet^®^ and Met52^®^) containing *M. brunneum*. In the horizontal transmission trials with GranMet^®^, healthy *P. japonica* adults were in contact with a single infected individual. In this case, a mortality of 100% was observed after 19 days, while in the trial with Met52^®^, the mortality was 30-65%. This result appeared promising and, if confirmed in extensive field trials, could represent a new tool for biological control strategies against the Japanese beetle. By contrast, Bosio et al. (65) found that Met52^®^ applied as a foliar spray against the adults did not differ from the control. To cope with *P. japonica* populations, Barzanti et al. (84) investigated the possibility of exploiting the presence of native strains of *Metarhizium* spp. in natural environments. For this purpose, the presence of *Metarhizium* species was analyzed in the soils of *P. japonica* infested areas. Four *Metarhizium* species were identified (*M. robertsii* J. F. Bisch., Rehner and Humber, 2009; *M. brunneum*; *M. guizhouense* Q.T. Chen and H. L. Guo, 1986, *M. lepidiotae* J. F. Bisch., Rehner and Humber, 2009) and used in virulence laboratory tests, with *M. robertsii* showing the best performance (84). The study confirmed the presence of native *Metarhizium* strains that can attack this invasive beetle and launched a debate on their future use in IPM programs. Entomopathogenic nematodes (Heterorhabditidae and Steinernematidae) have shown greater potential for the biological control of *P. japonica* than other natural biological agents (2). Therefore, EPNs, in association with other biological control agents, have been an important component of integrated pest management strategies in the US (2). The virulence of nematode species and strains against *P. japonica* grubs differed substantially (e.g., 85, 86) and contradicting results regarding the role of the larval stage on the susceptibility of *P. japonica* to EPNs, in laboratory studies, were reported. In Italy, for example, Paoli et al. (87) found that third-instar susceptibility to *Heterorhabditis bacteriophora* was higher in pre-overwintering than in overwintered larvae. Concerning EPNs at the beginning of the invasion in Italy, laboratory and field experiments were conducted with several native and commercial strains of *H. bacteriophora* and *Steinernema carpocapsae* Weiser, 1955 (Nematoda: Rhabditidae), in order to develop baseline data for a biological control approach for this outbreak (88). In the laboratory, *H. bacteriophora* strains caused higher mortality than *S. carpocapsae*, and the same results were obtained in micro-plot (2 × 6 m) field trials with an autochthonous strain of *H. bacteriophora*. Finally, in a large-plot (20 × 5 m) field trial, the commercial *H. bacteriophora* product (Larvanem) provided 46% larval mortality (88, 89) (**Figure 6**). This study highlighted that *H. bacteriophora* strains have good potential as biological control agents for the larvae of the invasive *P. japonica* in Northern Italy. This work, together with the restriction that only native EPN species can be field-released in Italy (EU Habitats Directive, art. 12, DPR 120/2003), has encouraged the search for local and better adapted EPN strains, especially in the two regions mostly infested by *P. japonica*: Piedmont (90) and Lombardy regions (91). Torrini et al. (90) reported that soils in the Ticino valley are rich in EPNs. The evaluation of all these EPN natural strains in laboratory assays confirmed that larval mortality was higher for pre-wintering than for post-wintering larvae, as already reported by Paoli et al. (87), and that *H. bacteriophora* natural strains are more efficient in controlling *P. japonica* larvae (88). Since native EPN isolates possess physiological traits that are adapted to local ecological conditions, the idea of supporting the ecosystem by propagating EPNs that thrive in their native soil could be considered as a basis for an eco-friendly approach to control this pest. In addition, a density-dependent response of EPNs to *P. japonica* has been shown since its introduction, indicating the great potential of these organisms as natural regulators of *P. japonica* populations (91). However, a decline in native scarab beetle populations has also been observed, indicating the generalist nature of soil EPNs (91). In addition to classic EPNs, other parasitic nematodes of *P. japonica* have been reported in Italy, such as the new species *Hexamermis popilliae* Poinar, 2017 (Nematoda: Mermithidae) (92) (**Figures 7A, B**) and *Oscheius myriophilus* (Poinar, 1986) (Nematoda: Rhabditidae) (93), both isolated from *P. japonica*. However, further studies are needed to assess the specificity of these nematodes and their possible use as biological control agents of *P. japonica*. In Italy, no parasitoids have been identified so far. However, it is noteworthy that the allochthonous parasitoid wasps *Tiphia vernalis* Rohwer, 1924, and *T. popilliavora* Rohwer, 1920 (Hymenoptera: Tiphiidae), which attack larvae, have established at low density in a wide range over the US. The same situation was observed for the fly *Istocheta aldrichi* (Mesnil, 1953) (Diptera: Tachinidae), which parasitizes adults (2) in the US. In Italy, generalist invertebrates (e.g., spiders) and vertebrate predators, (e.g., moles, birds) are under evaluation as potential natural enemies of *P. japonica*. Predators, especially ants, carabids, and spiders have caused high rates of mortality of *P. japonica* eggs and young larvae in the US (94, 95). The microsporidian *Ovavesicula popilliae* (Andreadis and Hanula, 1987) and the pathogenic bacteria, particularly *Bacillus* spp. and *Paenibacillus popilliae* (Dutky) Pattersson et al., 1999, causal agents of the milky disease, are present in the US (2, 96), but there are no data on their occurrence in Italy. In conclusion, considering that several EPN natural strains have been isolated in Italy and that an indigenous *H. bacteriophora* caused high mortality, EPNs seem to be particularly relevant for the control of this insect in Italy. EPNs can be combined with other methods such as insecticides (2) and EPF (97) to increase the pest control rate. However, several factors still limit the adoption of EPNs, such as the high cost, limited availability, short shelf-life, and formulations (2).

## 5 Conclusions

The accidental introduction of *P. japonica* into mainland Europe has led the Italian Plant Protection Organization to promote the development of multiple strategies to control the pest population and to deploy phytosanitary measures to prevent its spread. Previous knowledge from the US guided the first responses to the *P. japonica* invasion in Italy but, given the peculiarities of the local landscapes/crops and legislation restrictions on the use of chemicals and classical biological control agents, in-depth studies were needed for the development of management strategies to mitigate the impacts of *P. japonica* in newly invaded areas. Different control methods (i.e., chemical, physical, and biological control) have been developed and effective phytosanitary measures were taken to prevent the spread of the pest through the movement of plants, commodities, and vehicles from infested to pest-free areas. Despite ongoing regulatory efforts, *P. japonica* remains a threat and new areas have been colonized. As it was not possible to eradicate the pest and completely stop its natural spread, it is necessary to continue working on more effective and sustainable solutions in the context of an integrated management perspective.

In this context, the models developed to predict the phenology and spread of *P. japonica* can support the regulatory authorities in guiding decision-making toward rational and sustainable management of the pest.

As the use of insecticides in landscapes is increasingly restricted and registrations of some chemicals declined in the meanwhile, a higher level of protection could be achieved through an integrated approach that takes into account the specific landscape of an area and the occurrence of the main hosts of the pest. In addition to the use of spraying chemicals, which are effective under most conditions, the use of EPNs showed to be promising in lowering the larval population of *P. japonica* in treated areas and therefore can be a more eco-friendly or low-impact approach to control this pest. Moreover, the use of attract-and-kill devices turned out to be an effective strategy requiring minimal management effort with a high impact on adult populations. This strategy could be useful to slow down the spread rate of *P. japonica*. So far, EPF against larvae have only shown a significant effect in laboratory experiments, while results in the field seem to be limited. As far as parasitoids are concerned, there are no cases of natural and effective parasitism in Italy, and the possibility of importing parasitoids and other biological control agents from the native area of *P. japonica* is currently under investigation.

In conclusion, the knowledge of *Popillia japonica* acquired in Italy in recent years made it possible to better assess the population abundance, phenology, and spread. Moreover, these in-depth studies provided the basis for validating and directing control measures toward effective, sustainable, and environmentally sound management, as well as for developing new legislation for the control of this alien pest in the threatened European territory.

## Author contributions

This review was coordinated by the expert working group “Tavolo Tecnico *Popillia japonica*” MC, PG, GB, BC, EG, AA, NM, PR, and LM. All working group members contributed to the concept and structuring of the manuscript. MC, PG, GB, AA, NM, LM, GG, GM, GT, FP, and ABa wrote the first draft and all other authors (BC, AS, FL, GSa, GSp, EG, PR, and ABi) provided significant inputs into the text. All authors contributed to the article and approved the submitted version.
